# Supplementary material for: Non-falciparum malaria infection and IgG seroprevalence among children under 15 years in Nigeria, 2018
Source: Nat Commun. 2023 Mar 13;14:1360. doi: 10.1038/s41467-023-37010-0 (PMC10011577; doi:10.1038/s41467-023-37010-0)
Supplement: Supplementary file 1 — Supplementary Information [file 41467_2023_37010_MOESM1_ESM.pdf]

Non-falciparum malaria infection and IgG seroprevalence among children under 15 years in Nigeria, 2018

Supplementary Information

Supplementary Table 1. Tabulation for distribution of active infections by age of children for samples selected for PET-PCR assays.

Age (years)	Samples with Infecting *Plasmodium* species (n)							Age Total (n)
	Pf	Pm	Po	Pf/Pm	Pf/Po	Pm/Po	Pf/Pm/Po	
<1	18	0	0	1	0	0	1	20
1	33	1	0	4	0	0	0	38
2	60	1	1	8	1	0	0	71
3	76	0	0	12	3	1	0	92
4	79	1	0	18	1	0	1	100
5	79	2	1	13	8	0	0	103
6	81	0	1	13	0	1	1	97
7	66	2	0	12	5	0	0	85
8	72	1	0	21	5	0	0	99
9	50	0	0	7	1	0	2	60
10	63	0	1	16	1	0	0	81
11	30	1	0	7	3	0	0	41
12	50	2	0	6	0	0	0	58
13	39	0	0	5	1	0	1	46
14	32	2	0	6	1	0	0	41
**Total**	**828**	**13**	**4**	**149**	**30**	**2**	**6**	**1032**

Supplementary Table 2. Among children with any *Plasmodium* infection, multivariate adjusted analysis for risk of PET-PCR confirmed *P. malariae* and *P. ovale* mixed or mono-infection compared to *P. falciparum* mono-infections alone.

Variable	***P. malariae***[b] aOR (95% CI)	***P. ovale***[b] aOR (95% CI)
**Sex**		
Female	ref	ref
Male	0.85 (0.61, 1.19)	0.75 (0.39, 1.41)
**Age in years**		
<5	ref	ref
5–9	1.24 (0.83, 1.87)	2.24 (1.07, 5.41)*
10–14	1.29 (0.83, 2.03)	1.28 (0.46, 3.45)
**Wealth**		
Lowest	ref	ref
Second	1.17 (0.76, 1.80)	0.60 (0.25, 1.28)
Middle	0.59 (0.35, 0.96)*	0.37 (0.13, 0.86)*
Fourth	0.68 (0.38, 1.16)	0.41 (0.13, 1.05)
Highest	0.50 (0.20, 1.06)	0.07 (0.00, 0.52)*
**Place of residence**		
Rural	ref	ref
Urban	1.20 (0.79, 1.81)	1.38 (0.61, 2.88)
**Mosquito net coverage[a] (individual)**	1.20 (0.62, 2.23)	1.07 (0.33, 3.01)
**Mosquito net coverage[a] (community)**	0.57 (0.23, 1.24)	1.56 (0.35, 5.06)

*Statistically significant at alpha= 0.05

[a]Net coverage is defined as at least 1 net per 1.8 household members.

[b]Inclusive of *P. malariae* and *P. ovale* single-species infections, or mixed with *P. falciparum*

Supplementary Table 3. Seropositivity to PmMSP1, PoMSP1, and PvMSP1 among children age <15 years in Nigeria by state, 2018.

	**Children (n)**	***P. malariae* MSP1 *% (95% CI)***	***P. ovale* MSP1, *% (95% CI)***	***P. vivax* MSP1, *% (95% CI)***
**Total**	31,234	34.2 (33.3- 35.2)	12.1 (11.6- 12.5)	6.3 (6.0- 6.7)
North Central Zone				
Benue	932	31.3 (26.9, 35.7)	9.8 (7.3, 12.3)	4.7 (3.0, 6.5)
FCT*	234	19.7 (13.2, 26.3)	10.4 (5.9, 14.9)	4.6 (2.0, 7.2)
Kogi	504	31.3 (25.4, 37.1)	17.1 (12.2, 22.0)	10.5 (6.8, 14.1)
Kwara	424	28.3 (21.7, 34.8)	12.1 (8.4, 15.8)	9.6 (6.3, 13.0)
Nasarawa	425	36.7 (30.5, 43.0)	13.0 (9.1, 17.0)	6.2 (3.6, 8.8)
Niger	1290	32.0 (27.5, 36.5)	11.5 (9.3, 13.7)	6.3 (4.7, 7.9)
Plateau	775	32.1 (25.6, 38.6)	11.7 (9.0, 14.4)	6.6 (4.9, 8.4)
North East Zone				
Adamawa	855	41.3 (34.6, 47.9)	13.8 (9.6, 17.9)	6.2 (4.4, 8.1)
Bauchi	1420	41.7 (37.1, 46.3)	12.0 (10.0, 14.1)	8.0 (6.1, 9.8)
Borno	636	24.2 (14.3, 34.1)	6.4 (3.4, 9.3)	4.6 (2.8, 6.3)
Gombe	822	39.1 (33.8, 44.3)	9.9 (7.4, 12.5)	6.0 (4.2, 7.7)
Taraba	687	42.3 (36.7, 47.9)	14.5 (11.4, 17.5)	7.7 (5.2, 10.2)
Yobe	576	29.3 (22.3, 36.2)	9.0 (6.0, 12.0)	4.9 (2.8, 7.0)
North West Zone				
Jigawa	1374	39.5 (35.0, 44.1)	8.6 (6.8, 10.4)	6.2 (4.4, 8.0)
Kaduna	1816	40.5 (35.4, 45.7)	10.7 (8.9, 12.4)	6.8 (5.5, 8.1)
Kano	1828	29.4 (24.8, 33.9)	9.4 (7.7, 11.1)	4.1 (3.1, 5.1)
Katsina	1609	50.0 (44.3, 55.8)	14.5 (12.1, 16.9)	9.3 (7.5, 11.1)
Kebbi	862	46.8 (41.2, 52.4)	19.0 (15.2, 22.7)	17.6 (13.7, 21.4)
Sokoto	848	38.4 (33.8, 42.9)	15.8 (12.1, 19.5)	12.5 (9.6, 15.5)
Zamfara	371	27.6 (21.8, 33.4)	14.1 (9.8, 18.4)	9.2 (5.4, 13.0)
South East Zone				
Abia	645	27.0 (21.5, 32.5)	10.8 (8.2, 13.4)	3.0 (1.6, 4.5)
Anambra	881	16.0 (12.4, 19.5)	6.4 (4.8, 8.1)	1.7 (0.9, 2.5)
Ebonyi	691	57.6 (53.2, 62.1)	18.5 (15.1, 22.0)	7.5 (5.1, 9.9)
Enugu	678	32.2 (26.8, 37.5)	10.1 (7.3, 12.9)	3.0 (1.7, 4.2)
Imo	1089	28.1 (23.5, 32.8)	14.2 (11.5, 17.0)	2.5 (1.5, 3.5)
South South Zone				
Akwa Ibom	793	53.2 (47.9, 58.5)	22.3 (18.5, 26.0)	7.6 (5.5, 9.8)
Bayelsa	468	38.2 (31.3, 45.0)	14.4 (10.4, 18.4)	5.8 (3.6, 7.9)
Cross River	601	39.1 (33.8, 44.4)	11.5 (8.7, 14.3)	5.5 (3.6, 7.5)
Delta	911	31.2 (25.4, 37.1)	13.6 (10.4, 16.8)	6.3 (4.5, 8.2)
Edo	657	29.2 (24.0, 34.4)	13.1 (9.6, 16.7)	5.0 (3.3, 6.8)
Rivers	935	29.2 (23.7, 34.7)	14.3 (11.2, 17.3)	5.0 (3.2, 6.9)

South West Zone				
Ekiti	325	40.8 (33.3, 48.2)	17.9 (13.3, 22.5)	6.5 (3.7, 9.3)
Lagos	1671	5.2 (3.9, 6.5)	2.9 (2.1, 3.7)	1.7 (1.0, 2.4)
Ogun	601	20.5 (16.1, 24.9)	6.9 (4.3, 9.4)	3.6 (2.1, 5.2)
Ondo	540	41.1 (34.9, 47.2)	18.5 (15.4, 21.5)	9.5 (6.6, 12.5)
Osun	442	41.0 (33.7, 48.3)	12.8 (9.5, 16.1)	7.0 (4.3, 9.8)
Oyo	1018	35.7 (30.9, 40.6)	15.2 (12.8, 17.6)	7.3 (5.4, 9.3)

* Federal Capital Territory

Supplementary Table 4. Bivariate association models assessing socioeconomic risk factors for seropositivity to PmMSP1, PoMSP1, and PvMSP1.

Risk factor	Pm MSP1 OR (95% CI)	PoMSP1 OR (95% CI)	PvMSP1 OR (95% CI)
**Total household members**	1.02 (1.01, 1.03)*	1.02 (1.01, 1.03)*	1.02 (1.01, 1.03)*
**Own any livestock**	1.47 (1.37, 1.57)*	1.33 (1.23, 1.45)*	1.50 (1.34, 1.67)*
**Main roof material**			
Natural roof (no roof, mud, or hatch)	ref	ref	ref
Finished roofing	0.63 (0.57, 0.69)*	0.86 (0.76, 0.96)*	0.66 (0.58, 0.76)*
Rudimentary roofing	0.69 (0.54, 0.87)*	0.77 (0.58, 1.03)	0.80 (0.56, 1.14)
Other	0.58 (0.49, 0.70)*	0.93 (0.76, 1.14)	0.76 (0.58, 0.99)*
**Main wall material**			
Natural wall (no walls, dirt, cane/ palm)	ref	ref	ref
Finished walls	0.66 (0.56, 0.77)*	0.83 (0.68, 1.00)*	0.72 (0.56, 0.92)*
Rudimentary walls	1.27 (1.08, 1.49)*	1.13 (0.93, 1.37)	1.26 (0.99, 1.61)
Other	0.78 (0.55, 1.09)	0.97 (0.65, 1.44)	0.65 (0.37, 1.14)
**Air conditioner present**	0.47 (0.39, 0.58)*	0.47 (0.35, 0.63)*	0.39 (0.25, 0.60)*
**Electricity**	0.45 (0.41, 0.48)*	0.64 (0.59, 0.70)*	0.52 (0.46, 0.58)*
**Location of water source**			
Elsewhere	ref	ref	ref
In own dwelling	0.89 (0.79, 1.01)	0.91 (0.78, 1.05)	1.25 (1.04, 1.49)*
In own yard	1.01 (0.92, 1.12)	0.98 (0.87, 1.11)	1.06 (0.91, 1.24)

*significant at alpha= 0.05

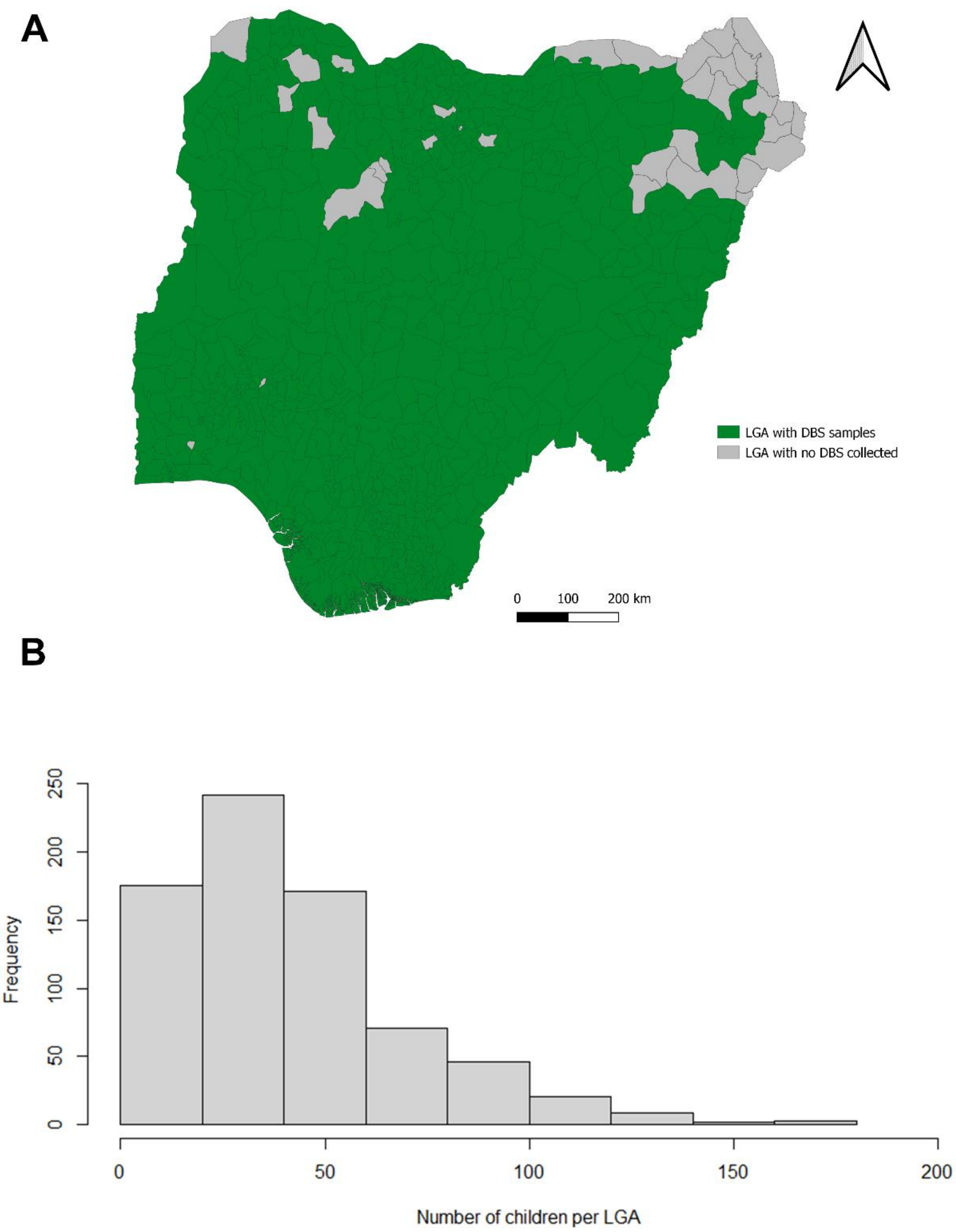

Supplementary Figure 1. (A) Map of Nigeria showing local government area (LGA) administrative units for which DBS from children age <15 years were collected and subsequent malaria data acquired. (B) Histogram of number of children <15y of age with DBS by LGA.

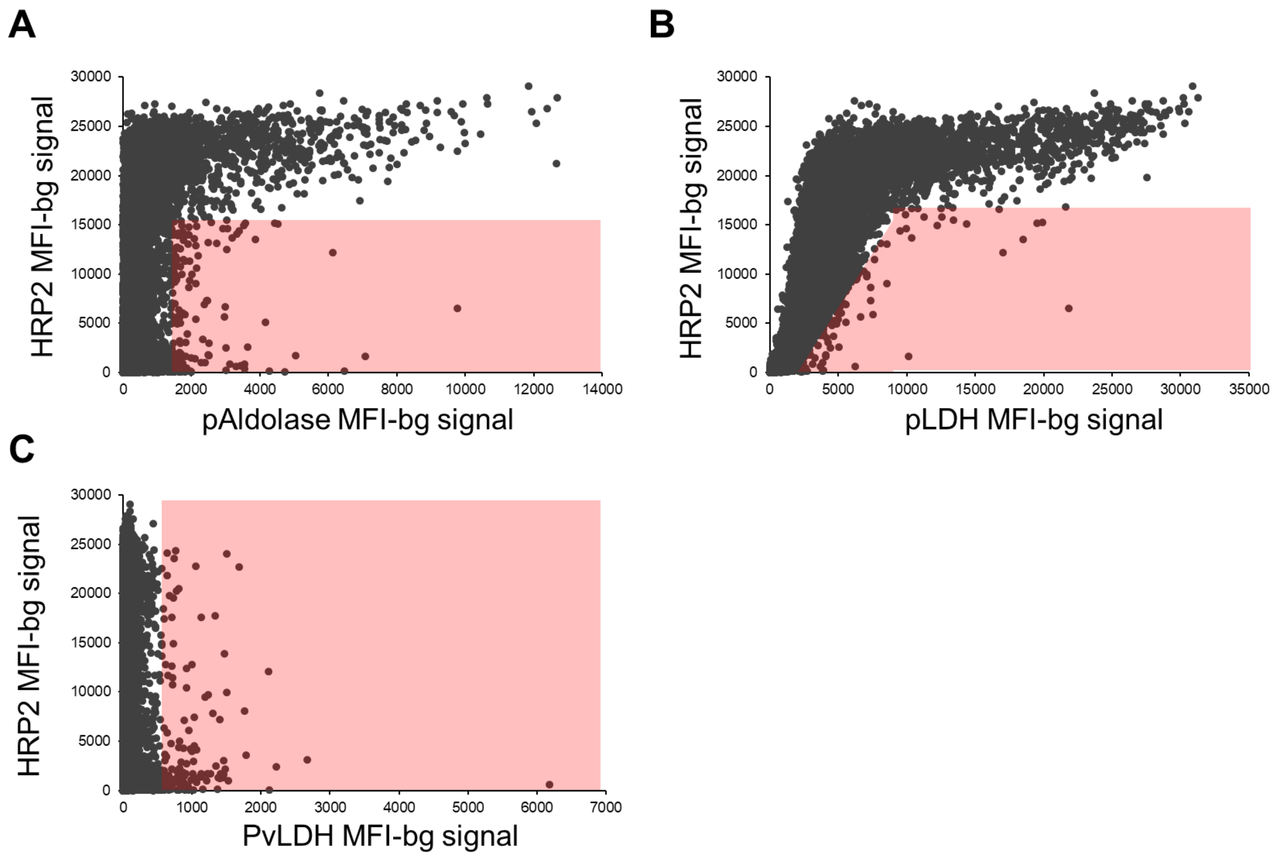

Supplementary Figure 2. Scatterplots of HRP2 assay signal in comparison to pan-*Plasmodium* aldolase (A), pan-*Plasmodium* LDH (B), and *P. vivax* LDH (C). Shaded area indicates DBS samples that were selected by low/absent HRP2 levels (A,B) or positivity to PvLDH (C) for later DNA assays.

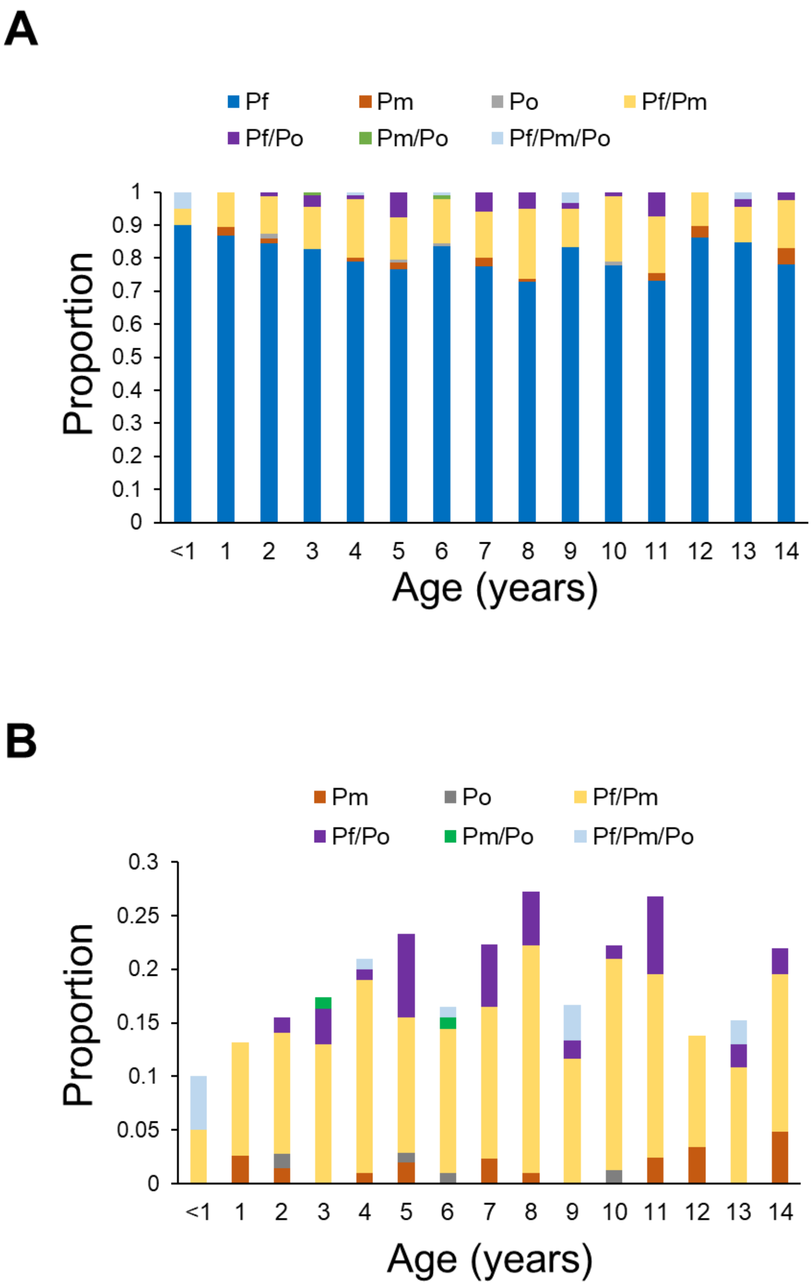

Supplementary Figure 3. Percentages of the species of *Plasmodium* infection by age in Nigerian children ages 0-14. (A) Proportions of infections from selected samples containing different *Plasmodium* species for all specimens and inclusive of *P. falciparum* mono-infections, and with *P. falciparum* mono-infections removed (B).

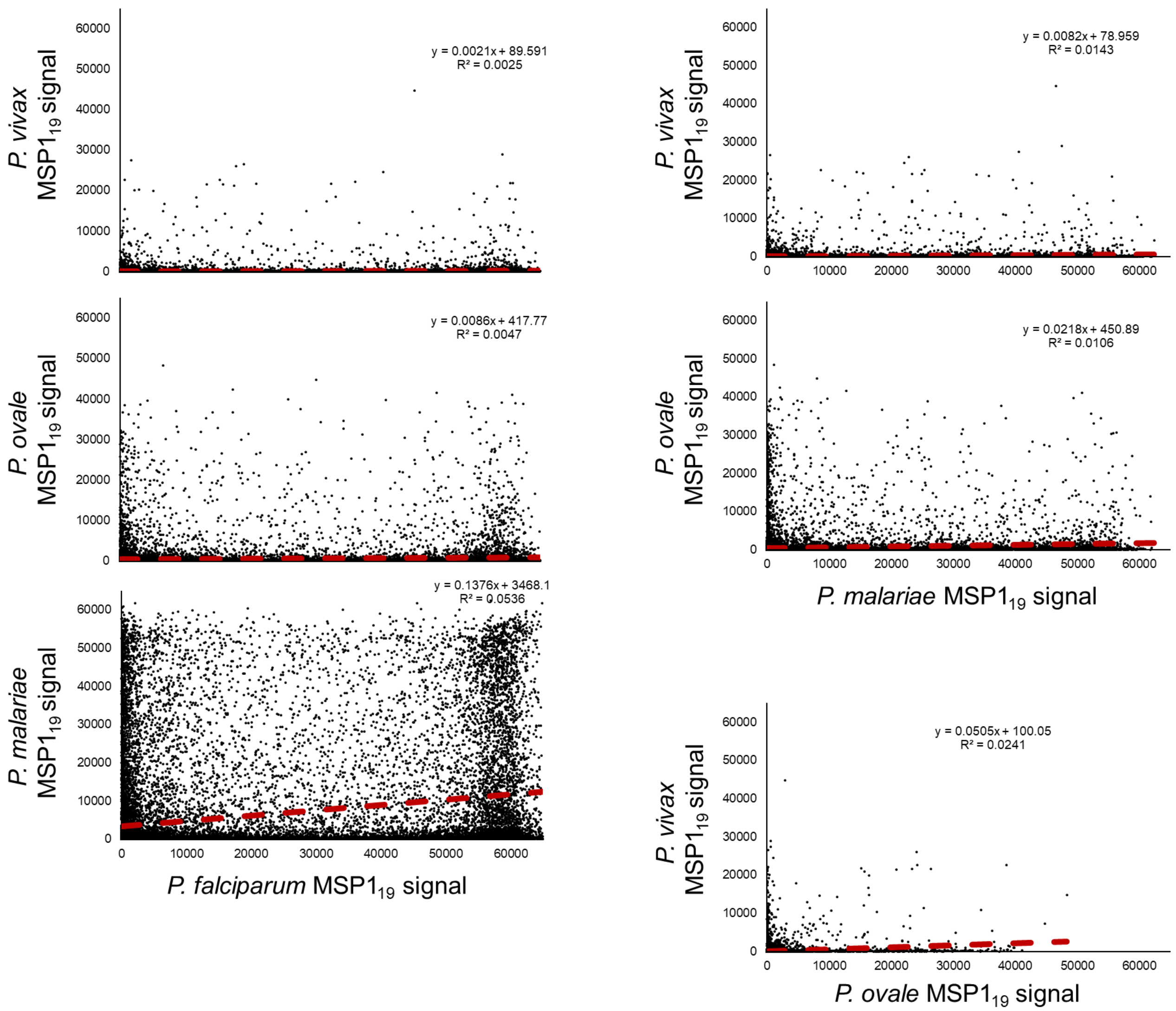

Supplementary Figure 4. Scatterplots of IgG assay signal with each species' MSP1-19kD antigen compared to all others. Red hashed line shows linear regression fitting, with inset text displaying model estimates.

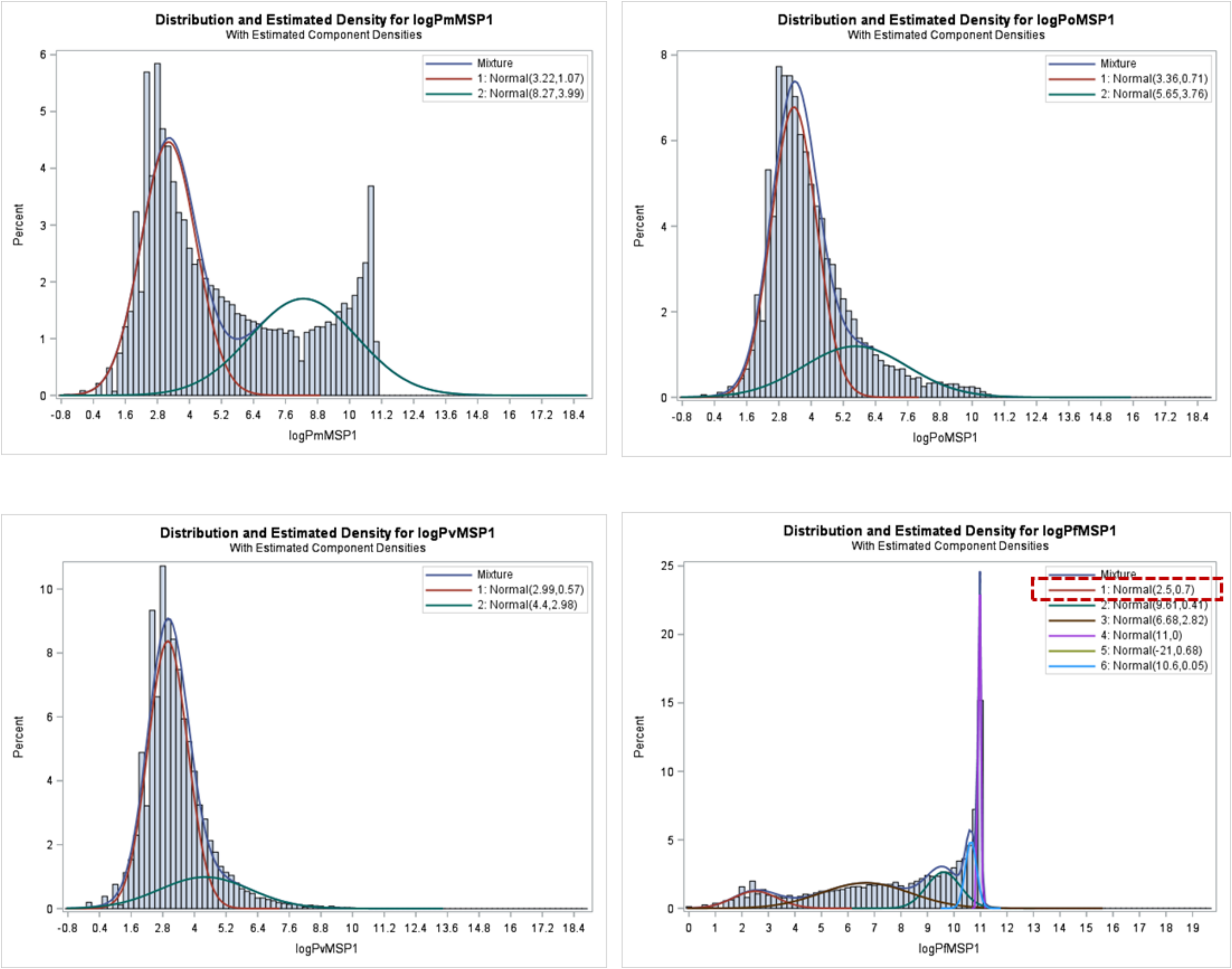

Supplementary Figure 5. Finite mixture models (FMM) of log-transformed assay signals for PmMSP1, PoMSP1, PvMSP1, and PfMSP1. For the two-component fittings (PmMSP1, PoMSP1, PvMSP1), the red density curve represents the putative seronegative population with estimates for distribution mean and variance in inset boxes. The PfMSP1 data needed to be fit to a six-component FMM in order to confidently ascertain the seronegative component – in red with parameter outputs in red hashed box.

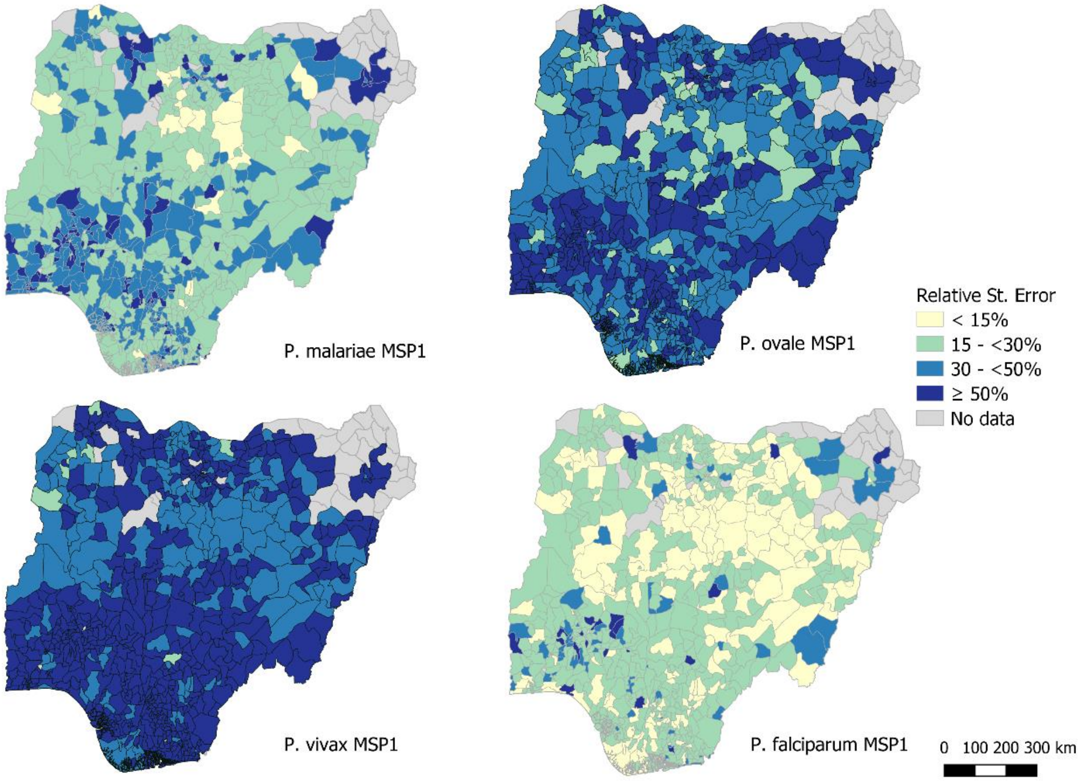

Supplemental Figure 6. Relative standard errors for seroprevalence estimates to MSP1 antigens by LGA.

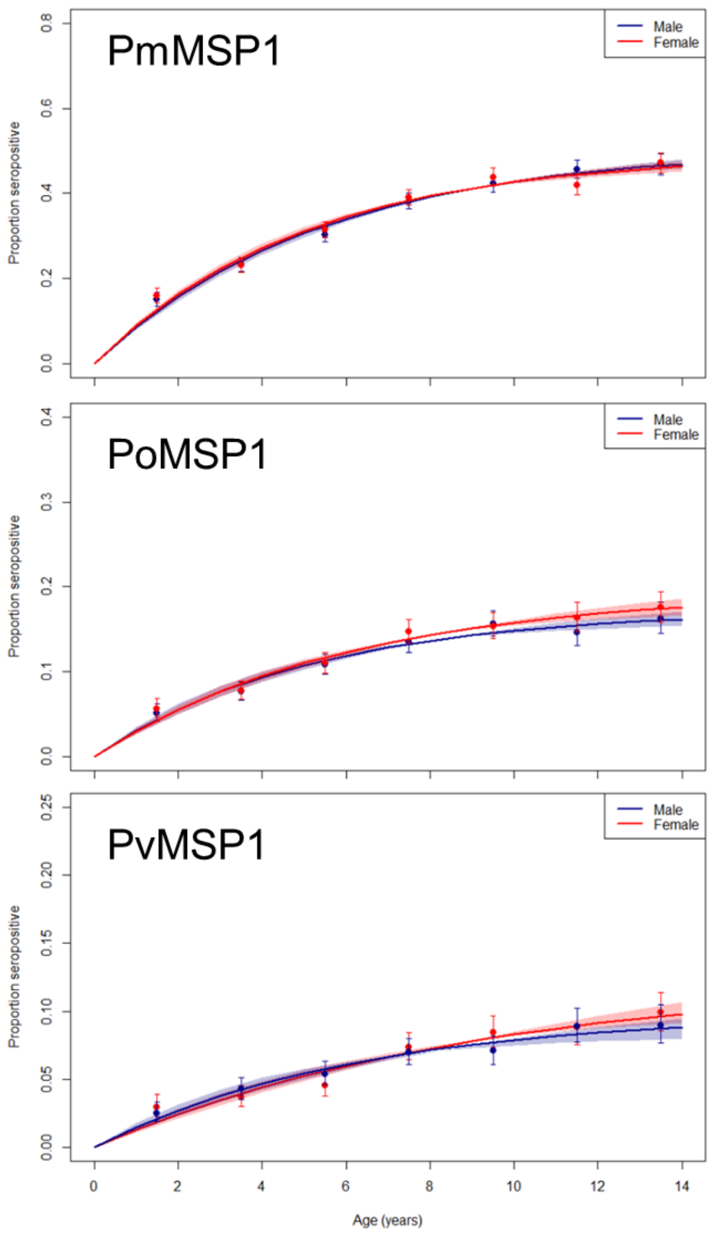

Supplementary Figure 7. Seroprevalence by age and sex for antibodies to *P. malariae*, *P. ovale,* and *P. vivax* MSP1 antigens. Dots represent the proportion seropositive and error bars represent the 95% confidence intervals for seropositivity for each age group.. Curves represent the fit of the catalytic conversion model, and the shading indicates the 95% credible interval of the model fit. For all plots, n=31,234 biologically independent samples.

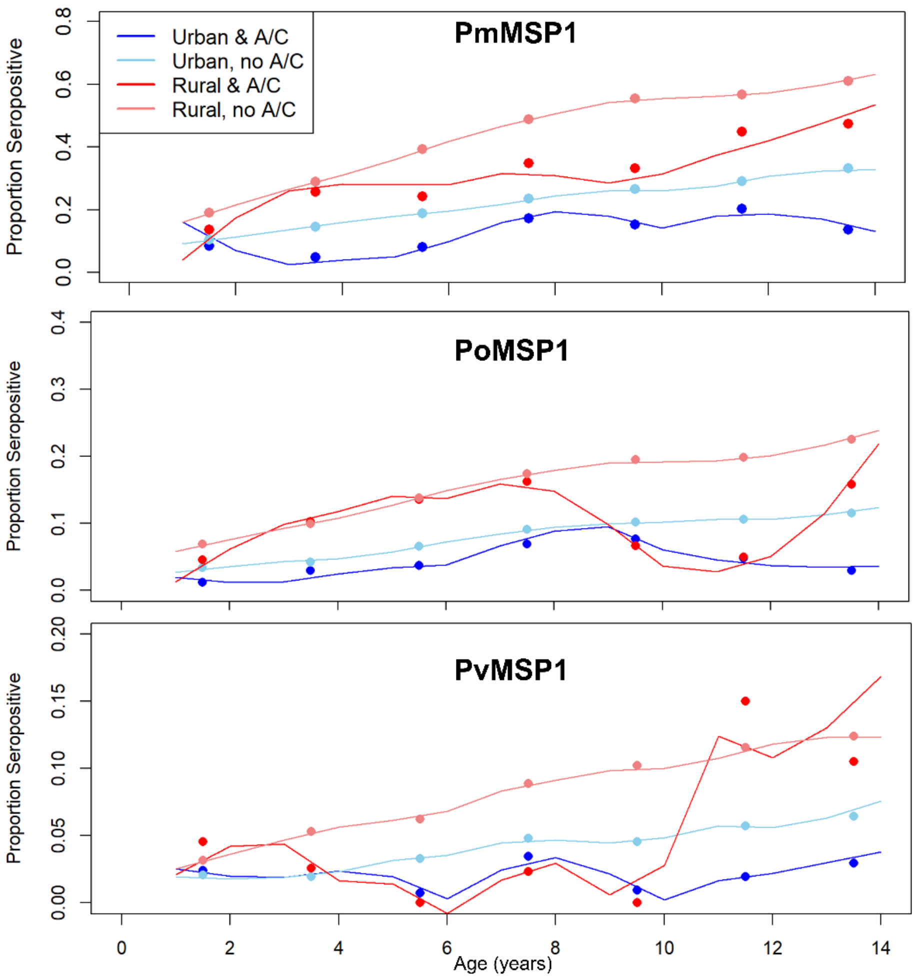

Supplementary Figure 8. Seroprevalence by age, urbanicity and presence of air conditioning (A/C) in the household for antibodies to *P. malariae*, *P. ovale,* and *P. vivax* MSP1 antigens. Points represent estimates for seroprevalence for each age category. Curves represent the fit of the LOESS regression.

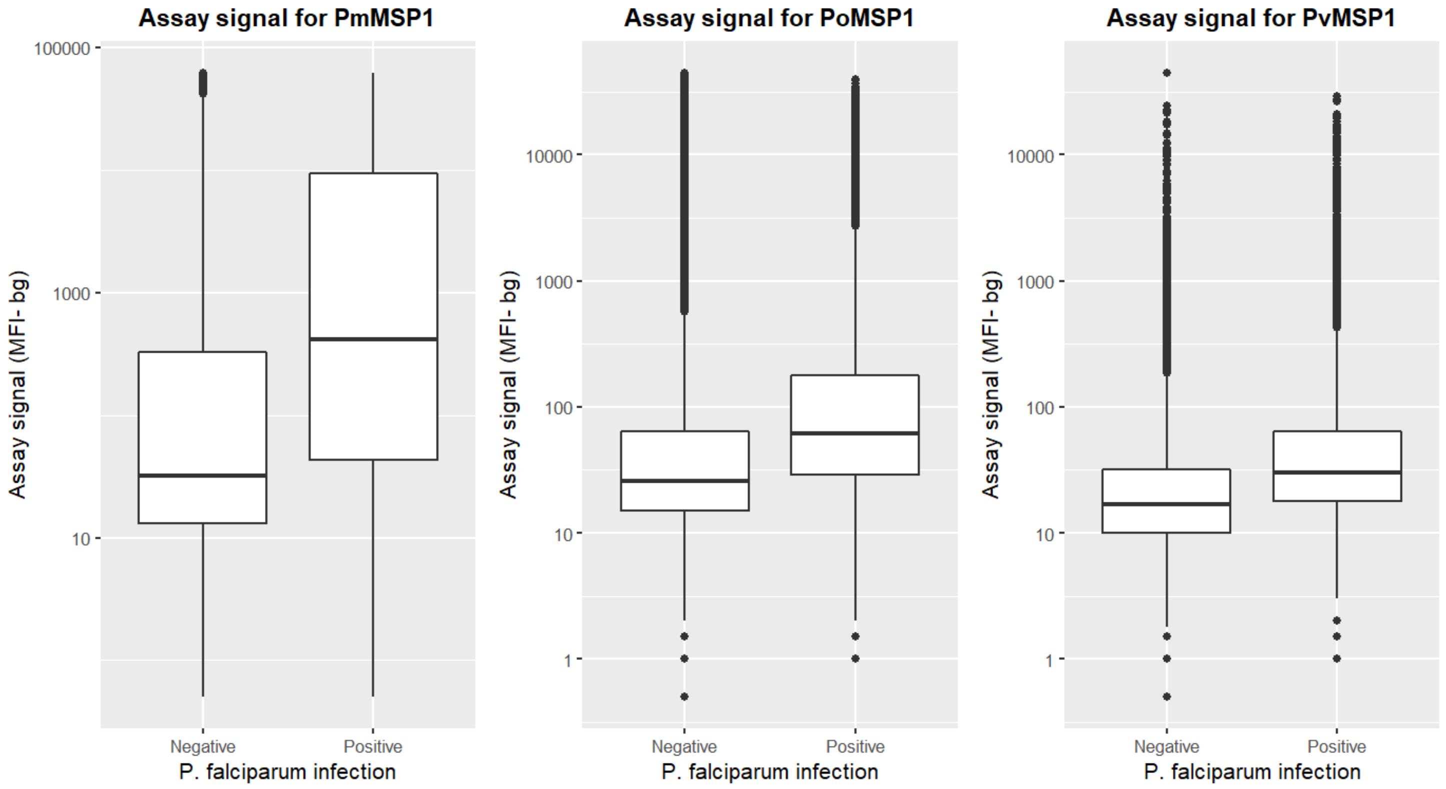

Supplementary Figure 9. Comparing the IgG assay signal for *P. malariae*, *P. ovale*, and *P. vivax* $MSP1_{19}$ targets by active *P. falciparum* infection. Boxes display interquartile range (IQR), with horizontal lines as median assay signals and whiskers extending 1.5x IQR above and below boxes and points at greater than 1.5x IQR. Each plot represents data from a total of 1,204 biologically independent samples that underwent PET-PCR analysis.

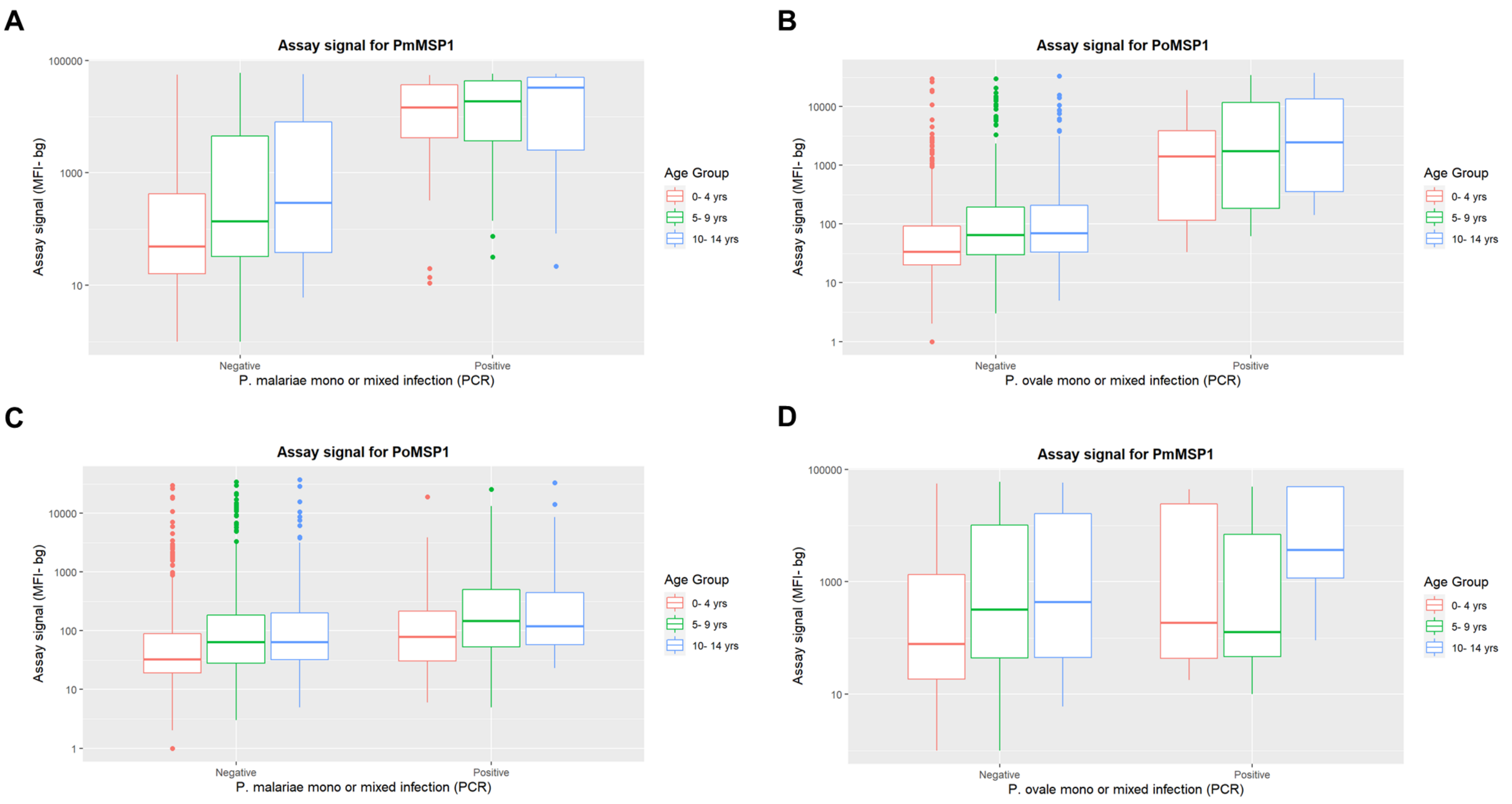

Supplementary Figure 10. Comparing MSP1 antigen assay signals by active malaria infection (PET-PCR positive for *P. ovale* and *P. malariae*) categorized by age. (A) PmMSP1 assay signal for children by *P. malariae* infection status. (B) PoMSP1 assay signal for children by *P. ovale* infection status. (C) PoMSP1 assay signal for children by *P. malariae* active infection status. (D) PmMSP1 assay signal for children by *P. ovale* active infection status. Boxes display interquartile range (IQR), with horizontal lines as median assay

signals and whiskers extending 1.5x IQR above and below boxes and points at greater than 1.5x IQR. Each plot represents data from a total of 1,204 biologically independent samples that underwent PET-PCR analysis.
