# Supplementary material for: Non-falciparum malaria infection and IgG seroprevalence among children under 15 years in Nigeria, 2018
Source: Nat Commun. 2023 Mar 13;14:1360. doi: 10.1038/s41467-023-37010-0 (PMC10011577; doi:10.1038/s41467-023-37010-0)
Supplement: Supplementary file 3 — Description of Additional Supplementary Files [file 41467_2023_37010_MOESM3_ESM.pdf]

Description of Additional Supplementary Files

File Name: Supplementary Data 1
Description: PET-PCR primers used in this study
