# Supplementary material for: Non-falciparum malaria infection and IgG seroprevalence among children under 15 years in Nigeria, 2018
Source: Nat Commun. 2023 Mar 13;14:1360. doi: 10.1038/s41467-023-37010-0 (PMC10011577; doi:10.1038/s41467-023-37010-0)
Supplement: Supplementary file 5 — Reporting Summary [file 41467_2023_37010_MOESM5_ESM.pdf]

nature portfolio

Corresponding author(s): Eric Rogier

Last updated by author(s): Jan 30, 2023

Reporting Summary

Nature Portfolio wishes to improve the reproducibility of the work that we publish. This form provides structure for consistency and transparency in reporting. For further information on Nature Portfolio policies, see our Editorial Policies and the Editorial Policy Checklist.

Statistics

For all statistical analyses, confirm that the following items are present in the figure legend, table legend, main text, or Methods section.

n/a	Confirmed	
☐	☒	The exact sample size (*n*) for each experimental group/condition, given as a discrete number and unit of measurement
☐	☒	A statement on whether measurements were taken from distinct samples or whether the same sample was measured repeatedly
☒	☐	The statistical test(s) used AND whether they are one- or two-sided<br>*Only common tests should be described solely by name; describe more complex techniques in the Methods section.*
☐	☒	A description of all covariates tested
☐	☒	A description of any assumptions or corrections, such as tests of normality and adjustment for multiple comparisons
☐	☒	A full description of the statistical parameters including central tendency (e.g. means) or other basic estimates (e.g. regression coefficient) AND variation (e.g. standard deviation) or associated estimates of uncertainty (e.g. confidence intervals)
☒	☐	For null hypothesis testing, the test statistic (e.g. *F*, *t*, *r*) with confidence intervals, effect sizes, degrees of freedom and *P* value noted<br>*Give P values as exact values whenever suitable.*
☐	☒	For Bayesian analysis, information on the choice of priors and Markov chain Monte Carlo settings
☐	☒	For hierarchical and complex designs, identification of the appropriate level for tests and full reporting of outcomes
☐	☒	Estimates of effect sizes (e.g. Cohen's *d*, Pearson's *r*), indicating how they were calculated

Our web collection on statistics for biologists contains articles on many of the points above.

Software and code

Policy information about availability of computer code

Data collection	Survey questionnaire data cleaning was conducted using Census and Survey Processing System (CSPro) version 7.7.2 and SAS v9.4 (SAS Institute, Cary, NC). Laboratory data collection occurred by Agilent Mx3005pro (for PCR) and Luminex xPonent v4.2 (for antigen and IgG detection) software.
Data analysis	SAS version 9.4, R version 4.1.1 (R Foundation for Statistical Computing, Vienna, Austria), Microsoft® Excel® version 2208. Analytical code is posted on Github at: https://github.com/cleonard297/nonPf_seroPCR_code

For manuscripts utilizing custom algorithms or software that are central to the research but not yet described in published literature, software must be made available to editors and reviewers. We strongly encourage code deposition in a community repository (e.g. GitHub). See the Nature Portfolio guidelines for submitting code & software for further information.

Data

Policy information about availability of data

All manuscripts must include a data availability statement. This statement should provide the following information, where applicable:

- Accession codes, unique identifiers, or web links for publicly available datasets
- A description of any restrictions on data availability
- For clinical datasets or third party data, please ensure that the statement adheres to our policy

DHS data are publicly available for download. NMS4 data are owned by the Government of Nigeria (GoN); requests for NMS4 data must be approved by GoN and all other NMS4 principal investigators. Inquiries should be directed to the corresponding author.

Human research participants

Policy information about studies involving human research participants and Sex and Gender in Research.

Reporting on sex and gender	Individual participants answered questionnaire question on sex based on their personal identification.
Population characteristics	NAIIS 2018 was a national HIV population-based household survey enrolling persons of all ages, and survey design was based on previous census data to provide population-representative estimates. Persons aged 1 month to 14 years were included in this analyses. No medical histories or information on past malaria exposure were collected.
Recruitment	Participants were approached at their place of residence for potential enrollment into this survey.
Ethics oversight	The National Health Research Ethics Committee of Nigeria and the Centers for Disease Control and Prevention

Note that full information on the approval of the study protocol must also be provided in the manuscript.

Field-specific reporting

Please select the one below that is the best fit for your research. If you are not sure, read the appropriate sections before making your selection.

☒ Life sciences ☐ Behavioural & social sciences ☐ Ecological, evolutionary & environmental sciences

For a reference copy of the document with all sections, see nature.com/documents/nr-reporting-summary-flat.pdf

Life sciences study design

All studies must disclose on these points even when the disclosure is negative.

Sample size	The target population was people 15-64 and children ages 0-14 years. Based on 2006 census data, the overall size and distribution of the sample is determined by analysis of existing estimates of national HIV incidence, sub-national HIV prevalence, and the number of HIV-positive cases needed to obtain estimates of VLS among adults 15-64 years for each of the 36 states and the FCT while not unnecessarily inflating the sample size needed. An equal-size approach was proposed with a sample size of 3,700 blood specimens from each Nigerian state. This representative sampling of the Nigerian populace is the largest household survey in Nigeria to date, and was sufficient for accurate and precise malaria estimates provided in this study.
Data exclusions	For the IgG analysis, data was excluded if failing the quality assurance step for non-specific binding. This led to 0.72% of all IgG datapoints being excluded. No other data was excluded from analyses.
Replication	Due to large sample size (>31,000 samples), antigen and IgG detection assays for individual samples were not repeated, and laboratory immunoassay data quality was confirmed through assay plate and internal well controls. All PCR assays were run in duplicate, and assay repeated for a sample if duplicate wells did not provide concordant positive/negative result.
Randomization	In this cross-sectional survey, participants were not pre-allocated into control and experimental groups. Within each Enumeration Area (EA), a random sample of 28 households was chosen to enroll participants.
Blinding	Laboratorians did not have access to individual-level data or characteristics. To report on prevalence estimates and risk factors in this study, all data was analyzed in an unbiased fashion without null hypothesis.

Reporting for specific materials, systems and methods

We require information from authors about some types of materials, experimental systems and methods used in many studies. Here, indicate whether each material, system or method listed is relevant to your study. If you are not sure if a list item applies to your research, read the appropriate section before selecting a response.

Materials & experimental systems

n/a	Involved in the study
☐	☒ Antibodies
☒	☐ Eukaryotic cell lines
☒	☐ Palaeontology and archaeology
☒	☐ Animals and other organisms
☒	☐ Clinical data
☒	☐ Dual use research of concern

Methods

n/a	Involved in the study
☒	☐ ChIP-seq
☒	☐ Flow cytometry
☒	☐ MRI-based neuroimaging

Antibodies

Antibodies used

anti-hIgG (Southen Biotech, 9042-08, C0013-RH25B); anti-hIgG4 (Southern Biotech, 9200-08, I2013-R895); anti-HRP2 IgG (ICLlabs, MPFG-55A, #8); anti-HRP2 IgM (ICLlabs, MPFM-55A, #8); anti-pAldolase (Abcam, ab207494, GR3336510-2); anti-PvLDH (Fitzgerald, 10-1334, M1709Pv2, #1938); anti-Plasmodium LDH (Fitzgerald, 10-P09I, M1209063, #3368)

Validation

Certificates of analysis available for each antibody on manufacturers' websites. Human Ig detection antibodies were previously validated by the manufacturer (Southern Biotech) with documentation available on their website. All antibodies for Plasmodium antigen detection had been previously utilized by our group and validated in preceding studies with DOIs: 10.1093/infdis/jiy525, 10.4269/ajtmh.19-0772. Anti-Plasmodium antibodies were validated with both purified recombinant antigens as well as blood samples from persons with active malaria infection to determine sensitivity and specificity against intended targets. Antibodies for immunoassays were verified by positive and negative panels before use in study, and assay plate performance ensured by positive and negative controls on each assay plate.
